# The effects of alpha lipoic acid (ALA) supplementation on blood pressure in adults: a GRADE-assessed systematic review and dose-response meta-analysis of randomized controlled trials

**DOI:** 10.3389/fcvm.2023.1272837

**Published:** 2023-10-24

**Authors:** Mahdi Vajdi, Nooshin Noshadi, Shirin Hassanizadeh, Atefeh Bonyadian, Hooria Seyedhosseini-Ghaheh, Gholamreza Askari

**Affiliations:** ^1^ Student Research Committee, Isfahan University of Medical Sciences, Isfahan, Iran; ^2^ Department of Clinical Nutrition, School of Nutrition and Food Sciences, Nutrition Research Center, Tabriz University of Medical Sciences, Tabriz, Iran; ^3^ Department of Clinical Nutrition, Faculty of Nutrition and Food Sciences, Nutrition Research Center, Shiraz University of Medical Sciences, Shiraz, Iran; ^4^ Nutrition and Food Security Research Center, Isfahan University of Medical Sciences, Isfahan, Iran; ^5^ Department of Community Nutrition, School of Nutrition and Food Science, Nutrition and Food Security Research Center, Isfahan University of Medical Sciences, Isfahan, Iran

**Keywords:** alpha lipoic acid, blood pressure, diastolic blood pressure, meta-analysis, systolic blood pressure

## Abstract

**Introduction:**

There have been various clinical studies on the effect of Alpha lipoic acid (ALA) supplementation on blood pressure (BP), but the findings from these are contradictory. Therefore, we performed a systematic review and dose-response meta-analysis to summarize the relation of ALA supplementation and systolic blood pressure (SBP) and diastolic blood pressure (DBP) in adults.

**Methods:**

A comprehensive search was conducted in Medline (PubMed), Embase, Scopus, and ProQuest up to July 2023. Randomized controlled trials (RCTs) evaluating the effect of ALA on SBP and DBP were included. The pooled weighted mean difference (WMD) of included trials was estimated using a random-effects model. The dose-dependent effect was also assessed.

**Results and discussion:**

A total of 11 RCTs with the participation of 674 patients were included. The result of the meta-analysis indicated that using ALA supplementation significantly reduced the SBP (WMD = −5.46 mmHg; 95% CI: −9.27, −1.65; *p* < 0.001) and DBP (WMD = −3.36 mmHg, 95% CI: −4.99, −1.74; *p* < 0.001). The ALA administrations significantly reduced SBP and DBP at the dosages of <800 mg/day, when administered for ≤12 weeks. The present meta-analysis revealed that ALA supplementation could exert favorable effects on SBP and DBP. Further well-designed studies with larger samples are needed to ascertain the long-term effects of ALA on BP.

**Systematic Review Registration:**

https://www.crd.york.ac.uk/prospero/display_record.php?RecordID=447658, identifier PROSPERO: CRD42023447658.

## Introduction

The chronic condition of hypertension (HTN) can lead to serious health complications and is recognized as one of the major significant health challenges on a global scale (1). HTN is a primary risk factor for heart failure, chronic kidney disease, cognitive impairment, dementia, and major cardiovascular events like stroke and heart attack (2). According to the World Health Organization, HTN will affect more than one billion individuals by 2025, with prevalence rates anticipated to rise from 26.4% to 29.2% (3, 4). The improvement of blood pressure (BP) in individuals is crucial from a clinical perspective, as a small reduction in BP could have a substantial impact on mitigating the burden of cardiovascular diseases (CVDs), resulting in significant public health benefits (5, 6). Despite the fact that over 90% of hypertensive cases are idiopathic (7, 8), according to the latest guidelines on the prevention and treatment of HTN, adopting healthy lifestyle habits (smoking cessation, diet and exercise) can delay or prevent the onset of high BP and can reduce the risk of CVDs (9). Furthermore, these lifestyle modifications can enhance the effectiveness of antihypertensive medications (9). The available evidence indicates that a diet with low-fat content and high in fruits and vegetables, combined with reduced sodium intake, has the potential to lower BP (10, 11). Clinical and experimental studies indicate that the incidence of certain CVDs, such as HTN, is linked to a rise in reactive oxygen species production (12). The use of antioxidant supplements taken orally could provide a cost-effective and beneficial alternative for the treatment of high BP (13, 14). Moreover, a Cochrane meta-analysis including 112,059 participants from 79 trials showed that omega-3 fatty acids from fish, which are high in eicosapentaenoic acid (EPA), and docosahexaenoic acid (DHA), have beneficial effects on cardiovascular health due to their anti-inflammatory properties (15).

Alpha-lipoic acid (ALA), which is synthesized by the liver and present in both animal and vegetable sources, is a potent mitochondrial antioxidant, functions through multiple pathways to support anti-inflammatory and antithrombotic processes, while also increasing vasodilation through nitric oxide mediation, leading to an improvement in endothelial function and a subsequent decrease in BP (16, 17). Further, ALA is thought to help with weight loss, lipid regulation, insulin sensitization, glucose regulation (18–20), as well as promote wound healing and reduce post-operative complications after cardiac surgery (21–23). Furthermore, ALA plays a crucial role in supporting enzymatic processes that metabolize nutrients into energy (24). It also has anti-inflammatory properties and lowers the risk of CVDs (25). However, the evidence for these uses is not conclusive and more studies are required to establish the effectiveness and safety of ALA supplements.

The effects of ALA supplementation on BP were also inconsistent across different studies; however, the exploration of the reason or mechanism for improving BP was not comprehensive. A number of animal and human studies have explored the impact of ALA on BP, with some suggesting that it may serve as a viable regulator of BP (26–30). According to Mohammadi et al. (31) ALA supplementation in patients with chronic SCI (spinal cord injury) significantly reduced systolic blood pressure (SBP) and diastolic blood pressure (DBP). Also, Pourghasem Gargari et al. (28) showed that supplementation of ALA decreases SBP and DBP in patients with rheumatoid arthritis. In contrast, another study conducted by Bobe et al. (30) has showed significant changes in SBP and DBP after long-term ALA supplementation. The results of another study also demonstrated that no significant change in SBP, and DBP after three months of supplementing with 800 mg of ALA (29). The effect of ALA supplementation on BP was also assessed in a systematic review in 2017. In this study, the effect of ALA supplementation on BP was evaluated similarly to the present study, but a meta-analysis, GRADE assessment, and dose-response analysis were not performed (32). Thus, given the current lack of consensus, we performed a systematic review and meta-analysis of published randomized controlled trials (RCTs) to assess the effects of ALA supplementation on SBP and DBP in adults. Furthermore, the current study used a GRADE assessment and a dose–response analysis, which improved the reliability of the results.

## Methods

This meta-analysis was conducted in accordance with the Preferred Reporting Items for Systematic Reviews and Meta-Analyses (PRISMA) guidelines (33) (Supplementary Table S1) and registered in the International Prospective Register of Systematic Reviews (PROSPERO) with the code CRD42023447658. The study protocol was approved by the ethics committee of Isfahan University of Medical Sciences (grant number: 1402161).

### Search strategy

The study was designed based on the following PICOS criteria: Population was the human model; intervention was ALA treatment; the comparison was control or placebo; outcomes were SBP and DBP; and study methodology was RCTs. We systematically searched electronic databases including Medline (PubMed), Embase, Scopus, and ProQuest to identify RCTs that examined the effects of ALA supplements on SBP and DBP from inception to July 2023. No limitation was considered for language or publication year. The combination of MeSH and nonMeSH terms were used as follows: “alpha lipoic acid” OR “alpha-lipoic acid” “ALA” OR “*α*-lipoic acid” OR “*α* lipoic acid” OR “thioctic acid” AND “systolic blood pressure” OR “systolic blood pressure” OR “SBP” OR “DBP” OR “blood pressure” (Supplementary Table S2). Moreover, manual screening was conducted for the reference lists of eligible articles.

### Study selection

The following criteria were used to select studies for inclusion: (1) RCTs with a parallel or crossover design; (2) assessed the effects of ALA supplementation on SBP and DBP. Moreover, all RCTs which supplemented another compound (drugs or supplements) along with ALA in both groups (control and intervention) were included as well; (3) designs that provide enough data to assess outcomes at baseline and after intervention; (4) participants aged 18 years or older. According to the following criteria, studies were excluded: (1) the absence of a control or placebo group; (2) lack of sufficient information for computing the measures; (3) trials involving children or pregnant women; (4) letters, conference abstracts, case reports *in vivo* and *in vitro* studies. If another compound or lifestyle intervention along with ALA was supplemented just in the intervention group and was not supplemented in the control group at the same time, and due to the confounding effect and in order to elucidate the definite role of ALA alone, those RCTs were excluded.

### Data extraction

Two authors (MV, NN) extracted the data independently from each of the included articles. A chief investigator (H S-G) evaluated any inconsistencies to reach a consensus. For each eligible study, the following data were collected: authors' first names, publication year, location and design of the study, study duration, ALA dosage, gender, mean age, the sample size in each group, and health conditions of participants.

### Quality assessment and certainty of evidence

An assessment of the methodological quality of the included studies was conducted using the Cochrane quality assessment tool in the following domains: (1) random sequence generation; (2) participant and personnel blinding; (3) concealment of allocation sequence; (4) outcome assessment; (5) selective outcome reporting; (6) incomplete outcome data; (7) other possible causes of bias (34). The quality items were divided into three groups: low risk, high risk, or unclear risk, based on their bias risk (34). In order to grade the overall certainty of evidence across the studies, GRADE (Grading of Recommendations, Assessment, Development, and Evaluation) Working Group guidelines were used (35). Evidence quality can be classified into four categories: low, moderate, and high, depending on the evaluation criteria (36).

### Statistical analysis

Our meta-analysis was conducted using Stata (StataCorp, College Station, TX, USA) version 14. Based on the means and standard deviations (SDs) reported for the control and intervention groups, we calculated the overall estimates (37). Weighted mean differences (WMD) and 95% confidence intervals (CIs) were used to measure treatment effects (38). The heterogeneity among studies was assessed using Cochrane Q and I-squared (*I*^2^) statistics, defining a significant heterogeneity as Cochrane Q <0.10 and/or *I*^2^ >50% (34). The fixed-effects model was selected when no significant heterogeneity was observed; otherwise, the random-effects model was applied. A subgroup analysis was conducted to detect heterogeneity based on the duration of intervention, dose of ALA supplementation, gender, health condition of participants, and sample size. An analysis of sensitivity was performed to determine the impact of each study on the overall effect size. Publication bias was evaluated using Egger's and Begg's tests with visual inspection of funnel plots. Moreover, a fractional polynomial model was also used to determine the non-linear effects of ALA dosage and treatment duration on SBP and DBP (39).

## Results

### Study selection

**Figure 1:**
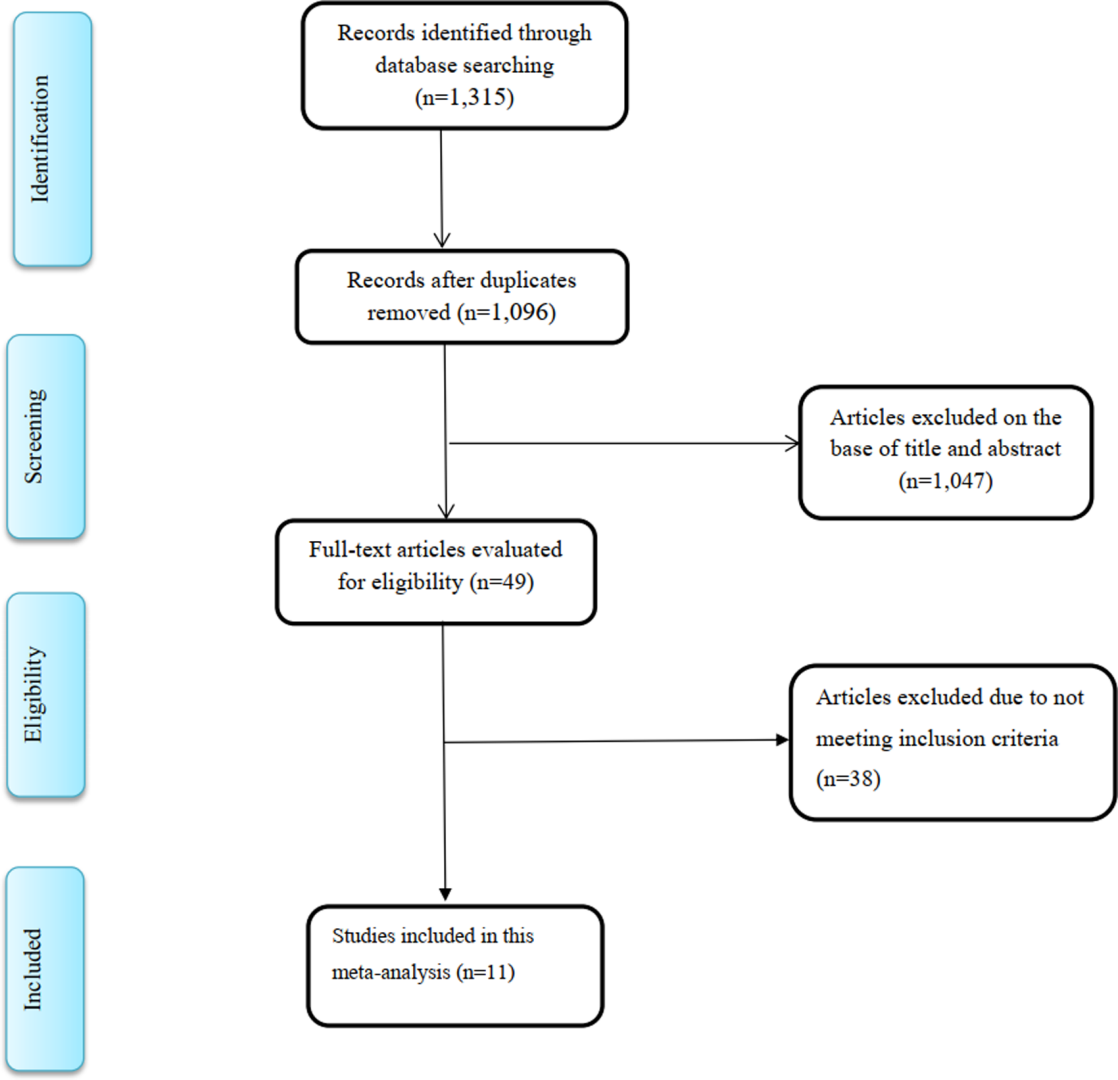
Flow diagram of study screening and selection process.

A total of 1,315 articles were recorded from the literature search. After removal of duplicates, 1,096 studies remained, of which 1,047 articles were excluded during the reviewing of titles and abstracts. After comprehensive full-text review of the remaining 49 studies, 38 studies were further omitted because the studies did not include the data necessary to analyze the findings in a meta-analysis. Finally, 11 RCTs (17, 28, 30, 31, 40–46), with 13 intervention arms, were selected for the current meta-analysis (Figure 1).

### Characteristics of included studies

**Figure 2:**
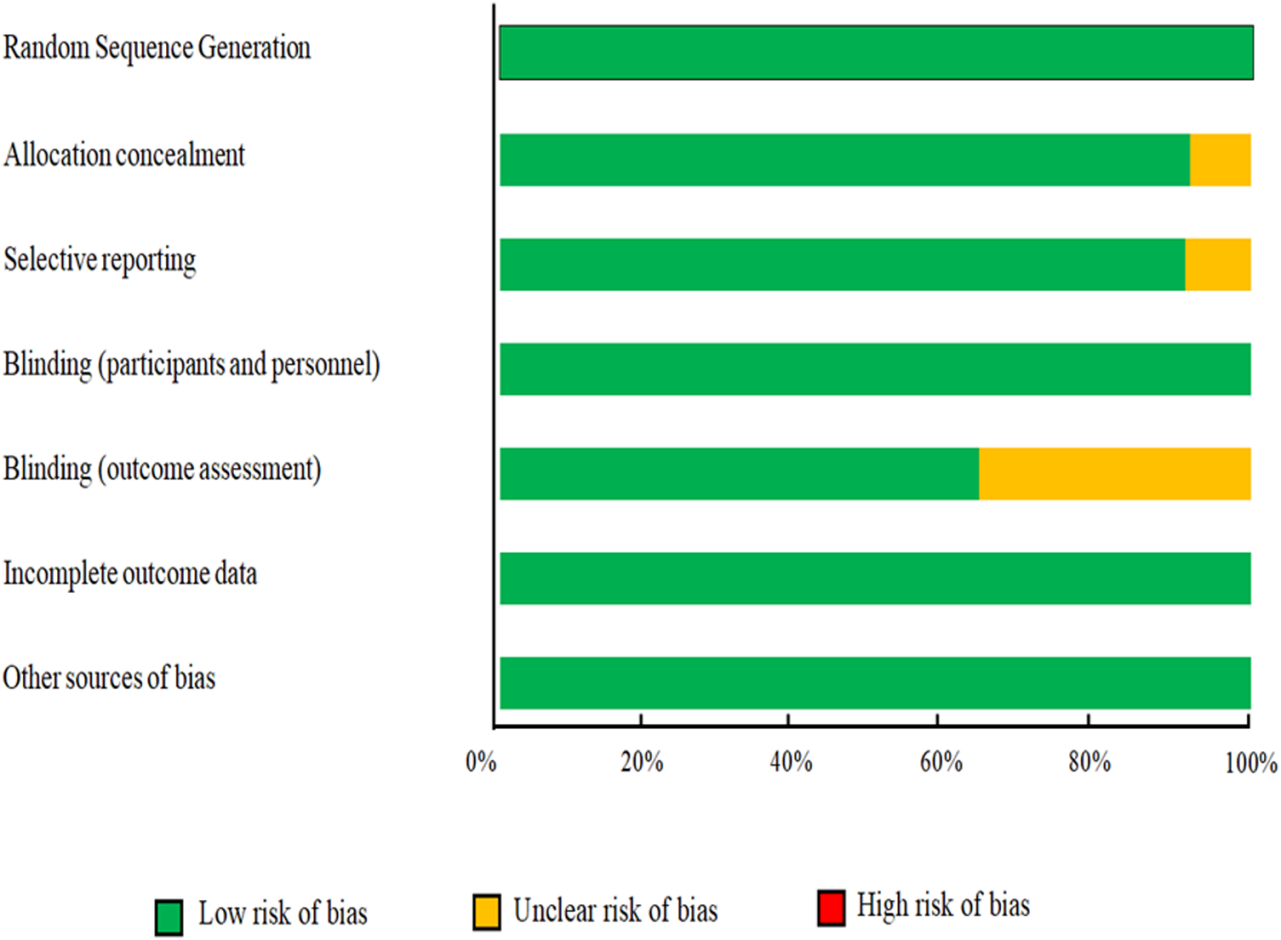
Results of risk of bias assessment for trials included in the current meta-analysis on the effects of ALA supplementation on blood pressure.

The characteristics of the included trials are summarized in Table 1. There were 674 participants included (cases = 346 and control = 328) in these RCTs. Eligible trials were published between 1997 and 2021. The studies were performed in Iran (28, 31, 40, 42), United States (41, 45, 47), Mexico (17), Italy (43), Korea (44), and Germany (46). The intervention duration of selected RCTs ranged from 8 to 48 weeks, with a sample size ranging from 12 to 155 participants. Of the eleven studies included in this meta-analysis, eight involved participants of both genders, while two focused exclusively on female subjects (28, 43) and one on male subjects (31). ALA supplementation dosage varied between 600 mg/day to 1,800 mg/day. Participants in included studies were patients with rheumatoid arthritis (28), type 2 diabetes mellitus (T2DM) (17, 45), takotsubo cardiomyopathy (43), pre-diabetics (41), stroke (42), non-insulin dependent diabetes mellitus (NIDDM) (46), SCI (31), overweight, and obesity (40, 44, 47). The quality assessment of the included trials according to the Cochrane Collaboration's risk of bias tool is presented in Figure 2. Overall, the included trials had a low risk of bias. Most trials revealed adequate quality for key factors. All of the included studies had a low risk of bias for random sequence generation, blinding (participants and personnel), incomplete outcome data and other sources of bias. Appropriate blinding (outcome assessment) was observed in 64% of included studies. Finally, the risk of selective reporting and allocation concealment was unclear in one study.

**Table 1:** Characteristics of included studies in the meta-analysis.

First author	Year, country	Subjects	Participants		Gender	Mean Age		Mean BMI		Design	Supplement	Comparator	Dose (mg/d)	Duration (week)	Baseline mean (SBP/DBP)		Medications	Adjustments	Main results
			Int	Con		Int	Con	Int	Con						Int	Con			
Nasiri et al.	2021, Iran	Overweight	21	21	B	34.86	34.67	27.78	27.57	RDBPC	ALA + probiotic	Probiotic	600	16	(140.40/91.27)	(140.17/91.28)	–	–	Significant reduction in SBP and DBP in the ALA group
Bobe et al.	2020, United States	Overweight	31	33	B	38	40	34.8	34.4	RDBPC	(R)- ALA	Placebo (Placebo type unclear)	600	24	(127/75)	(125/74)	–	–	No differences in SBP and DBP between groups
Mendoza-Núñez et al.	2019, Mexico	T2DM	42	38	B	63	64	28.69	28.96	RCT	ALA	Placebo (Microcrystalline cellulose)	600	24	(124/78)	(126/77)	Glibenclamide/metformin	–	No significant difference in SBP and DBP between groups
Gosselin et al.	2019, United States	pre-diabetics	12	12	B	47.1	47.1	33.4	33.4	RDBPC	ALA	Placebo (cellulose)	600	4	(130.2/87)	(130.2/87)	–	–	No significant difference in SBP and DBP between groups
Mohammadi et al.	2018, Iran	Stroke	33	34	B	62.33	64.23	–	–	RDBPC	ALA	Placebo (wheat flour)	600	12	(133.18/84.24)	(132.94/86.02)	–	Baseline values, energy intake, and weight	Significant reduction in SBP and DBP in the ALA group compared with the placebo group
Raffaele et al.	2016, Italy	TCM	22	21	F	63.7	63.9	27	27.6	RDBPC	ALA	Placebo	600	48	(122/81.3)	(123/82.2)	Beta-blockers, ACEi/ARB, Aspirin, and Statin	–	No significant change in SBP and DBP between groups
Gargari et al.	2015, Iran	RA	33	32	F	36.09	38.28	29	29.02	RDBPC	ALA	Placebo (Maltodextrin)	1,200	8	(121.59/117.65)	(77.04/72.69)	–	Baseline values	Significant reduction in SBP and DBP between groups
Mohammadi et al.	2015, Iran	SCI	28	30	M	39	36.8	27.77	28.02	RDBPC	ALA	Placebo (wheat flour)	600	12	(126.43/123.50)	(87.85/82.50)	–	Baseline covariates and changes in weight	Significant reduction in SBP and DBP between groups
Koh et al.	2011, Korea	Obesity	82	73	B	41.4	40.7	33.3	33.1	RDBPC	ALA	Placebo (lactose and cellulose)	1,800	20	(137.48/84.67)	(131.95/79.95)	Antihypertensive/hypoglycemic agents/lipid-lowering medications	–	No significant difference in SBP and DBP between groups
Lukaszuk et al.	2009, United States	T2DM	13	7	B	56	53.14	33.8	33.3	RDBPC	R- ALA	Placebo (Microcellulose)	600	13	(127.6/78.3)	(123.9/76.3)	Anti-diabetic medication and/or insulin, anti-hypertensives, anti-hyperlipidemic, anti-inflammatories, and…	–	No significant differences in blood pressure between the groups
Ziegler et al.	1997, Germany	NIDDM	29	27	B	57.9	58.6	–	–	RDBPC	ALA	Placebo (Placebo type unclear)	800	16	(144/81.4)	(144/85.3)	Insulin treatment/Oral anti-diabetic agents	Baseline values	No significant difference in SBP and DBP between groups

ALA, alpha-lipoic acid; BMI, body mass index; DBP, diastolic blood pressure; SCI, spinal cord injury; TCM, Takotsubo cardiomyopathy; RA, Rheumatoid arthritis; RDBPC, randomized double-blind placebo-controlled; RCT, randomized controlled trial; T2DM, SBP, systolic blood pressure; Type 2 Diabetes mellitus, NIDDM, Non-insulin-dependent diabetes mellitus; M, male; F, female; B, both; Int, intervention; Con, control.

### GRADE assessment

The overall certainty of the evidence for the effects of ALA supplementation on SBP and DBP is presented in Supplementary Table S3. The quality of the evidence for SBP was graded as “moderate” after being downgraded for publication bias. Meanwhile, the quality of the evidence for DBP was graded as “high”.

### Findings from meta-analysis

#### Effect of ALA supplementation on SBP

**Figure 3:**
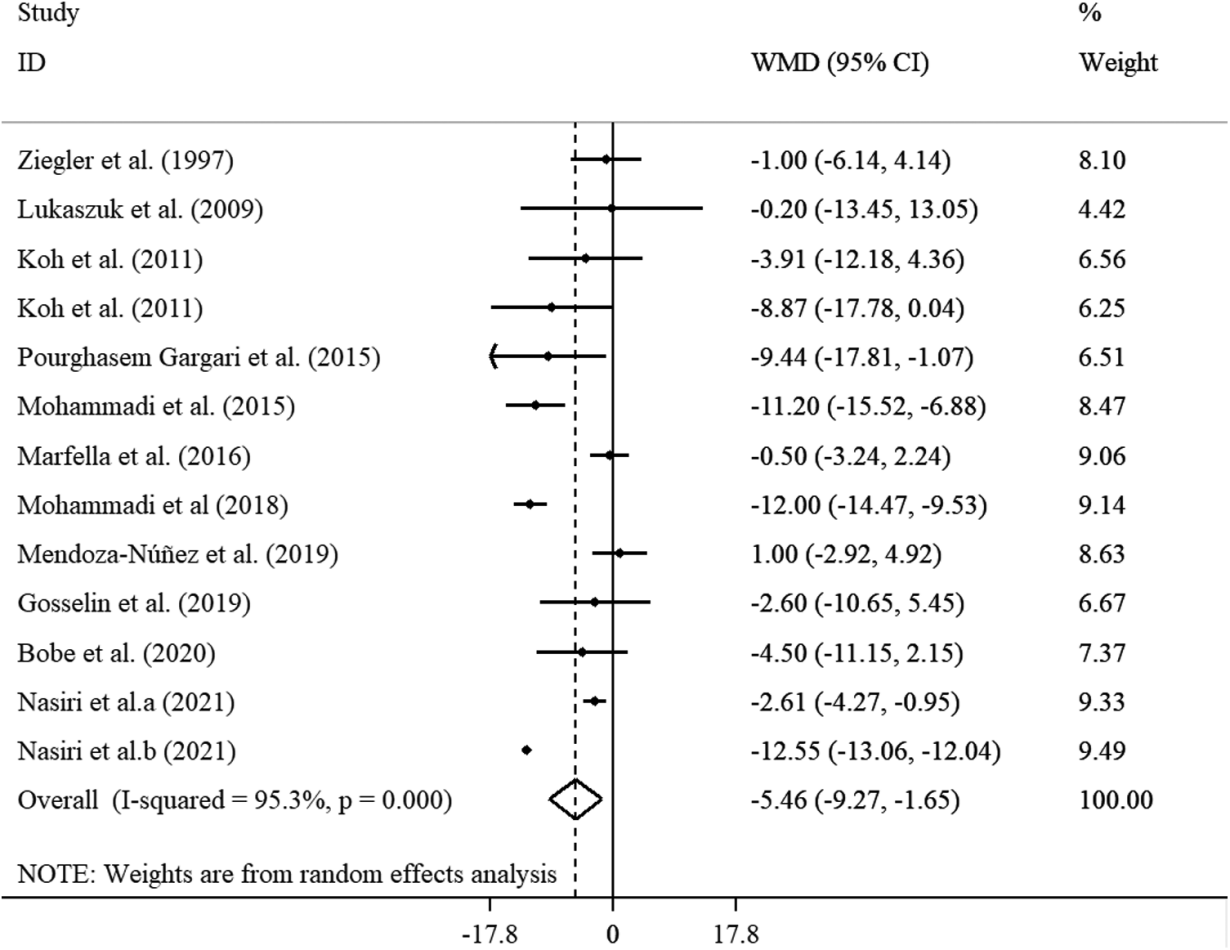
Forest plot illustrating weighted mean difference and 95% confidence intervals for the impact of ALA on SBP.

The effects of 11 studies, containing 13 effect sizes that measured SBP levels after ALA supplementation (cases = 346 and control = 328), demonstrated a significant reduction in SBP levels (WMD = −5.46 mmHg; 95% CI: −9.27, −1.65; *p* < 0.001) (Figure 3). As there was a significant heterogeneity (*I*^2^ = 95.3%; *p* < 0.001), we performed subgroup analysis and found that sample size, supplementation dosage, baseline BMI, duration of follow-up, participants' mean age, health status, and gender could explain between-study heterogeneity. Reductions in SBP levels were more pronounced in subjects aged <45 years, those with a baseline BMI of 25–29.9 kg/m^2^, in trials that prescribed <800 mg/day ALA, as well as in studies with ≤12 weeks of duration, in studies with sample size <60, and those performed on subjects with overweight and obesity (Supplementary Table S4).

#### Effect of ALA supplementation on DBP

**Figure 4:**
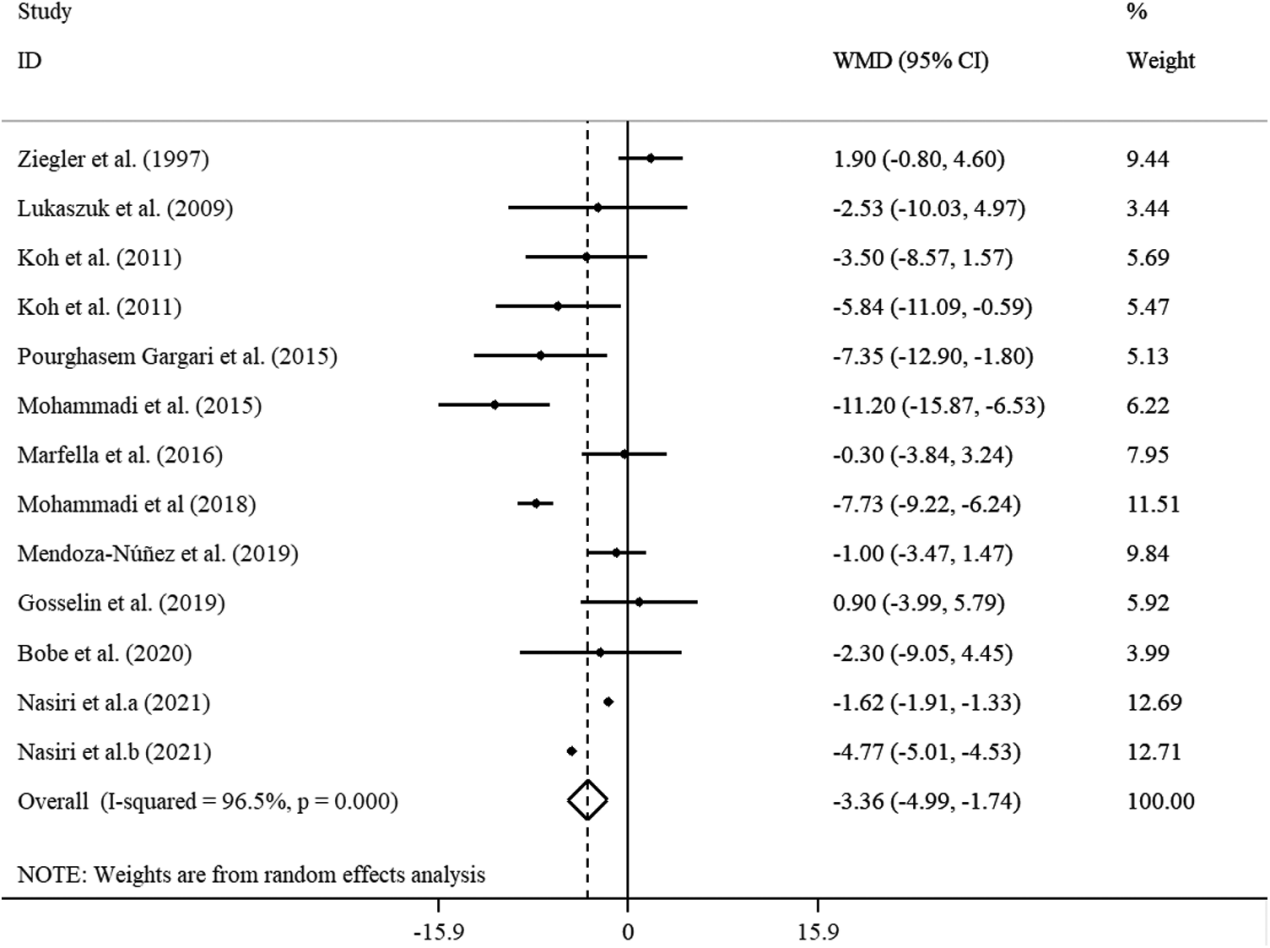
Forest plot illustrating weighted mean difference and 95% confidence intervals for the impact of ALA on DBP.

The analysis of the 11 studies with 13 effect sizes (cases = 346 and control = 328) revealed a significant reduction in DBP (WMD = −3.36 mmHg, 95% CI: −4.99, −1.74; *p* < 0.001), with high heterogeneity (*I*^2 ^= 96.5%; *p* < 0.001), after ALA supplementation (Figure 4). In the subgroup analysis, we found that the sample size, supplementation dosage, baseline BMI, duration of follow-up, participants' mean age, health status, and gender explained this heterogeneity. Reductions in DBP levels were more pronounced in subjects aged ≥45 years, those with a baseline BMI of 25–29.9 kg/m^2^, in trials that prescribed <800 mg/day ALA, as well as in studies with ≤12 weeks of duration, in studies with sample size ≥60, and those performed on both genders (Supplementary Table S5).

#### Meta-regressions and non-linear dose-response meta-analyses

**Figure 5:**
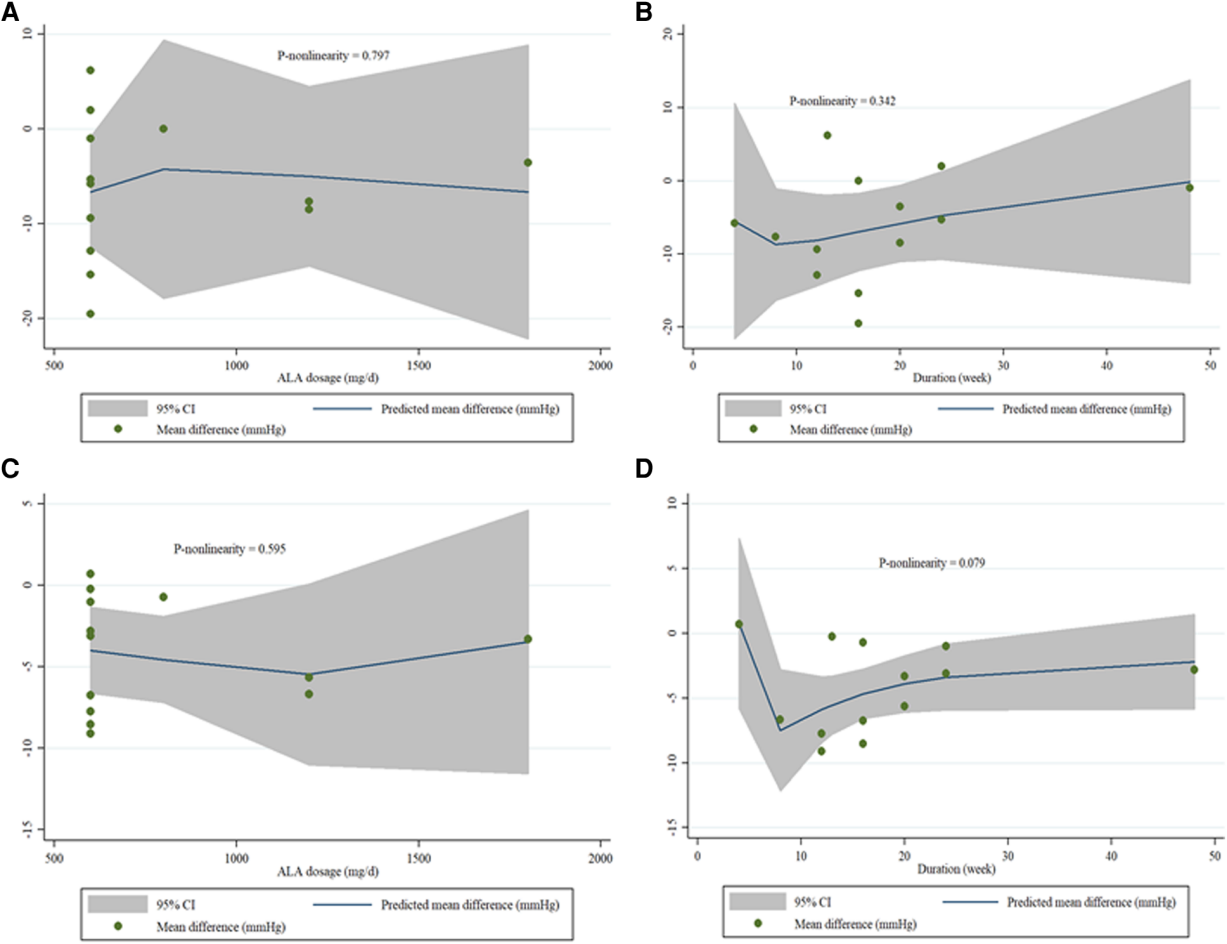
(**A–D**). Dose-response relations between and ALA dosage (mg/d) and duration of treatment (week) and mean difference in SBP (**A,B**), and DBP (**C,D**).

A non-linear dose-response analysis was carried out between the dose and duration of ALA supplementation for SBP and DBP. In the non-linear dose-response analysis, we did not observe a significant effect of dose and duration of ALA supplementation on SBP (*P* non-linearity = 0.797; 0.342), and DBP (*P* non-linearity = 0.595; 0.079) (Figures 5A–D). We performed a meta-regression analysis to examine the potential relationship between a decrease in SBP and DBP levels with dose and duration of ALA supplementation. The analysis did not show any significant association between the dose and duration of intervention with changes in SBP and DBP (Supplementary Figures S1A–D).

#### Sensitivity analysis

The sensitivity analysis was also performed to evaluate the effect of each individual trial on the pooled effect size by removing each trial in turn. The sensitivity analysis demonstrated that the calculated overall effect sizes for SBP and DBP were not substantially changed after removing each study individually (Supplementary Figures S2A,B).

#### Publication bias

Visual inspection of the funnel plot revealed no evidence of publication bias in studies assessing the effect of ALA supplementation on SBP and DBP (Supplementary Figures S3A,B). There was no evidence of publication bias for studies examining the effect of ALA on DBP (Begg's test: *p *= 0.669 and Egger's test: *p *= 0.910). Nevertheless, result of Egger's test showed a significant publication bias in studies that reported the effect of ALA on SBP (Begg's test: *p *= 0.502; and Egger's test: *p *= 0.023). However, the trim-and-fill analysis yielded findings similar to the original.

## Discussion

This systematic review and meta-analysis comprehensively evaluated the effects of supplementation of ALA on BP in adults. The results support the positive effect of ALA administration on lowering SBP and DBP levels compared with the control group. Sensitivity analysis shows that the calculated overall effect sizes for SBP and DBP did not change significantly after each individual study was removed, suggesting that the study results are robust and reliable, and do not depend on a single study.

Subgroup analyses showed that the direction of the effects of ALA was generally the same in the subgroups based on sample size, study duration, participant age, and initial BMI for SBP and DBP. However, we found a significant subgroup difference in participants' health status, the results shows that ALA supplementation was more effective in lowering SBP and DBP in obese and overweight participants and had no significant effect on prediabetes patients. Previous studies indicated that obesity may affect BP through several mechanisms. For example, obesity has been associated with high angiotensin levels II, increased aldosterone secretion, increased renal tubule sodium uptake, and consequently, increased BP (48). Thus, the reason why supplementation of ALA was more effective in obese and overweight patients may be due to the weight-reducing effects of ALA in addition to the antihypertensive effect of ALA (49). In regards to the doses of supplementation of ALA, although both doses of ALA (more than and less than 800 mg/day) were found to lower SBP, the results were more significant at the dose of less than 800 mg/day. As for DBP, only a dose of less than 800 mg/day significantly lowered DBP. However, the credibility of the dose-based subgroup should be interpreted with caution due to the small number of studies conducted with doses greater than 800 mg/day. It is recommended that further studies be conducted with different doses to make these results clearer and more reliable. In addition, the results of our meta-regression showed no significant relationship between dose and duration of ALA supplementation with changes in SBP and DBP. The difference between findings of subgroup analysis and meta-regression can be explained by the fact that in the subgroup analysis, the dose and duration of ALA supplementation were entered in classified forms and analyzed, while in meta-regression analysis, dose and duration of ALA supplementation were entered in continuous form.

In this meta-analysis, ALA reduced SBP by 5.46 mmHg and DBP by 3.36 mmHg. It is an antioxidant that may help with CVDs, diabetes, and inflammation (21–23). However, the evidence for its effectiveness is inconclusive. Although ALA is known for its antioxidant effects, it may not be very beneficial for reducing BP. Moreover, it also has some potential drawbacks, such as gastrointestinal, urological, nervous, or allergic complications or skin rashes in some individuals who use it (50, 51). Consequently, the benefits of ALA are not well-established and may vary depending on the duration, dose, and individual factors. Moreover, there was controversy in previous meta-analyses investigating the effect of antioxidant supplementation on BP (52–55). A study conducted by Guan et al. (52) reported that vitamin C supplementation significantly reduced SBP and DBP in patients with essential HTN. In contrast, resveratrol administration had no significant effect on SBP or DBP in a systematic review and meta-analysis, although significant results were observed in studies with resveratrol dosages of more than 300 mg daily and in diabetics (54). In a meta-analysis by Emami et al. (55) vitamin E supplements were found to reduce only SBP and not DBP. In the context of cardioprotection, a majority of studies have indicated that a higher body status of omega-3 fatty acids, particularly eicosapentaenoic acid (EPA), and docosahexaenoic acid (DHA), can significantly reduce the risk of CVDs (56, 57). However, it's worth noting that some studies have reported only a marginal reduction in coronary heart disease (CHD) deaths and CHD events with increased intake of EPA and DHA. Furthermore, an increment in the intake of *α*-linolenic acid has been associated with a slightly reduced risk of cardiovascular events and arrhythmia (15). A lower risk of CVD has also been associated with higher intakes of omega-3 fatty acids, EPA, and DHA (15, 58) by modulating CVD risk factors, such as BP (59, 60). According to a dose-response meta-analysis, omega-3 fatty acids lower BP in doses between 2 and 3 grams per day. In groups at high cardiovascular risk, omega-3 fatty acid intakes above the recommended 3 g/d may be associated with additional benefits (61). Therefore, while the benefits are clear, the extent to which these antioxidants contribute to cardioprotection may vary.

The effect of ALA supplementation on lowering SBP and DBP was previously investigated in a review study. However, meta-analysis and dose-response analyses were not performed in this review. It was concluded in this review that since most of the included studies did not show a significant effect of ALA supplementation on BP, associations between ALA and BP might not be significant (32). A variety of mechanisms may be involved in the action of ALA supplementation on SBP and DBP, for example, ALA may help reduce BP by inhibiting the reduction of SIRT3 (sirtuin 3), hyperacetylation of superoxide dismutase 2 (SOD2), and overproduction of reactive oxygen species (ROS) in mitochondria (62). It also increases endothelial nitric oxide and decreases angiotensin-converting enzyme activity (59, 63). The suppression of the renin–angiotensin system may be related to the activation of Peroxisome proliferator- activated receptor gamma (PPAR-*γ*) (64). Activation of PPAR-*γ* inhibits adhesion cascades and harmful inflammatory events in the vasculature. Thus, HTN may be treated by direct regulation of endothelial function and anti-inflammatory mechanisms in the vasculature triggered by PPAR-*γ* activation in humans (65, 66). Another mechanism for lowering BP is related to matrix metalloproteinases (MMPs) (67). MMPs alter smooth muscle tone, which can lead to a vicious cycle as BP increases (68, 69). However, further research should be conducted to elucidate the other mechanisms associated with the antihypertensive effect of ALA.

### Implications for practice

In this meta-analysis, ALA supplementation improved SBP and DBP. However, clinical targets are point to note in RCTs. Although the threshold for a clinically significant reduction in BP depends on individual circumstances, such as the patient's age and health status, one study reported that such a significant reduction can be considered to be at least 5 to 10 mm Hg for SBP compared to baseline or 3 to 5 mm Hg for DBP (70). In addition, this reduction may be associated with a lower risk of CVD and other health problems. In this meta-analysis, the researchers observed a reduction of −5.46 mm Hg and −3.36 mm Hg for SBP and DBP, respectively, clearly demonstrating the clinically significant effects of the study. As this supplement has not yet been approved by the FDA, further, larger, and longer studies are necessary to reach a more conclusive conclusion. In addition, these results cannot be applied to other health conditions that were not included in this study.

### Implications for research

Future studies should be conducted with a homogeneous population on a large scale. It is also imperative that confounding factors like absorption capacity, dietary adherence, lifestyle factors, and storage be taken into account when designing an appropriate RCT. The cost-benefit effects of ALA supplementation on improving BP should also be considered. Lastly, for determining the appropriate dosage of ALA, dose-escalation studies are also needed.

### Strengths and limitations

A number of strengths were identified in this meta-analysis: first, subgroup analyses were conducted based on several confounders; second, sensitivity analyses were carried out to ensure the results were robust; and third, the GRADE method was used to determine the degree of certainty. Nevertheless, some limitations must be considered. It was found that the included studies differed in terms of dosage, study duration, participants' age, and health status. Secondly, the studies included were heterogeneous. To identify the source of heterogeneity, we conducted subgroup analyses based on different variables. Third, it was not possible to consider the baseline levels of ALA in determining the effects of ALA on BP. Fourth, since our study included individuals with various health conditions, we could not identify which type of high BP (primary or secondary) is better treated with ALA. The effects of ALA supplementation on patients with primary and secondary HTN should be examined separately in future studies.

## Conclusion

A significant reduction in SBP and DBP levels was observed after ALA consumption. Further clinical studies are needed to evaluate the effects of ALA supplementation on different health conditions, in different doses, in different countries, and over longer periods of time, to confirm our findings.

## Data Availability

The raw data supporting the conclusions of this article will be made available by the authors, without undue reservation.
